# A theoretical design of evanescent wave biosensors based on gate-controlled graphene surface plasmon resonance

**DOI:** 10.1038/s41598-021-81595-9

**Published:** 2021-01-21

**Authors:** Ruey-Bing Hwang

**Affiliations:** 1 Institute of Communications Engineering, College of Electrical and Computer Engineering, National Chiao Tung University, Hsinchu, 30050 Taiwan; 2 Center for mmWave Smart Radar Systems and Technologies, National Chiao Tung University, Hsinchu, 30050 Taiwan

**Keywords:** Computational science, Sub-wavelength optics

## Abstract

A surface plasmon resonance (SPR) sensor based on gate-controlled periodic graphene ribbons array is reported. Different from the conventional methods by monitoring reflectivity variations with respect to incident angle or wavelength, this approach measures the change in SPR curve against the variation of graphene chemical potential (via dynamically tuning the gate voltage) at both fixed incident angle and wavelength without the need of rotating mirror, tunable filter or spectrometer for angular or wavelength interrogation. Theoretical calculations show that the sensitivities are 36,401.1 mV/RIU, 40,676.5 mV/RIU, 40,918.2 mV/RIU, and 41,160 mV/RIU for analyte refractive index (RI) equal to 1.33, 1.34, 1.35 and 1.36; their figure of merit (1/RIU) are 21.84, 24, 23.74 and 23.69, respectively. Significantly, the enhancement in the non-uniform local field due to the subwavelength graphene ribbon resonator can facilitate the detection in redistribution of protein monolayers modeled as dielectric bricks.

## Introduction

The emergence of the respiratory virus COVID-19 outbreak allows us to know the unprecedentedly urgent need of a rapid and accuracy diagnostic test method. Among the known diagnostic techniques, label-free optical biosensors provide high sensitivity and robustness solutions. Most importantly, such a biosensor can be integrated into a compact device and enables the possibility of bedside (or point-of-care) testing^1^. Optical biosensors monitor variations of the scattering characteristics of incident light including intensity, phase, wavelength, and angle due to interaction between the target analyte and bioreceptor such as nucleic acid sequences, proteins, antibody, enzymes, and etc. Optical fiber with a tapered small core for single-mode operation designed to serve as a high sensitivity RI sensor was reported^2^. The resonant waveguide gratings were extensively reviewed^3^, particularly including applications of sensors for detecting the RI change on the waveguide grating surface.

Surface plasmons are collective oscillation of the free electron gas density on the surface of noble metals or graphene. Through the electromagnetic wave coupling between photon and plasmon, the excitation of SPPs (surface plasmon polaritons) is present with its field exponentially decaying (evanescent wave) along the direction perpendicular to the surface^4–9^. Additionally, enhanced absorption of surface plasmons on a periodic array of graphene ribbons for TM-polarization (the magnetic field is along the ribbon axis) was reported^10–13^. Specifically, the obvious optical absorption due to the SPR on graphene ribbons, particularly on its fundamental resonant mode, was demonstrated^10^.

Moreover, the sensed medium attached to the position where SPR occurs strongly affects the resonance effect. Such a phenomenon has been widely applied in the design of biosensors for detecting the change in RI of an analyte. To mention a few, the literature focused on SPR sensors, which were designed for detecting chemical and biological species, based on the unlocalized surface plasmons propagating along wave-guiding structures were intensively reviewed^14^. A nanodevices for detection and real-time monitoring of biomolecular events using SPR and SPR imaging were thoroughly reviewed; the signal modulation schemes including angle, wavelength, amplitude and phase were discussed, as well as their coupling mechanism^15^. The SPR imaging techniques incorporating wavelength scanning based on solid-state wavelength filter were reviewed from the perspective of detection speed, sensitivity and portability^16^. RI sensor using long-range surface plasmon mode, which exhibits symmetric field pattern, was shown to have high sensitivity compared with conventional SPR ones due to low loss^17^. The plasmonic nanograting was employed to tailor the dispersion of plasmonic response; more specifically, the enhancement in quality (Q) factor further improves the figure of merit (FOM) of the biosensor^18^. The nanoplasmonic biosensor plays an important role in optical sensors because of its high sensitivity to the change in RI. More specifically, the biomolecular recognition elements on the surface of biosensors recognize and capture analyte in a liquid sample, causing an increase in the average RI on the sensor surface; several approaches for detecting the RI change were reported^15,19–23^. An effective design approach for achieving the high FOM of a guided-mode resonance sensor made of a grating-waveguide was numerically studied^24^. Furthermore, by adding a dielectric thin film having a high real part of permittivity for dramatically improving the sensor sensitivity was reported^25^. In their study, the guided-wave SPR structure can enhance the evanescent field at the interface between silicon and cover. Additionally, by spin-coating the GeSe nanosheets on the surface of noble metal (Au), a SPR sensor can significantly enhance its sensitively^26^.

Regarding the biosensors made of graphene ribbons, the researchers detected multiple SPR absorption bands on graphene ribbons by changing the graphene chemical potential^27^. A transmission type SPR-based sensor made of graphene nanoribbons array in infrared (IR) wavelength range was proposed to detect the wavelength shift of the resonant transmission dip due to the RI change of the analyte medium placed on the sensor surface^28^. A graphene-based tunable mid-IR biosensor was demonstrated on the protein characterization including both complex RI extraction and vibration fingerprints. The gate-controlled self-biasing graphene ribbons array is dynamically tuned to selectively excite the local SPR mode at different frequencies^29^.

This research reports the computational design of a biosensor for RI sensing at mid-IR wavelengths. Specifically, the SPR modes supported by graphene nanoribbons are excited by evanescent wave rather than plane wave. The excitation of SPR modes in the nanoresonators (nanoribbons) enables the local-field enhancement around the slit (the region between two adjacent ribbons) edges. The placement of analyte on the surface of nanoresonators strongly affects the resonant condition of surface plasmons, particularly on the fundamental mode. As is well known, the wavelength- and angle-interrogation are the two major approaches for detecting the RI change due to a shift in SPR-curve. Significantly, in this research, graphene chemical potential scan is employed to replace the sophisticated wavelength modulation or incident angle scan. By electronically tuning the gate voltage (dc) to change the chemical potential (Fermi energy) in graphene, one can detect the smallest resolvable RI change of an analyte through the shift in SPR curve (against the variation of gate voltage). Furthermore, the tunable gate voltage can be provided by a high-resolution digital-to-analog converter (DAC) equipped with an amplifier. Furthermore, a periodic dielectric bricks array was employed to model discrete sensing mediums such as protein monolayers. The perturbation on SPR curve due to their redistribution and variation of surface percentage coverage (concentration) were also examined. Such a mechanism can be a potential candidate for designing a cost effective biosensor with acceptable performance parameters.

## Results and discussion

**Figure 1:**
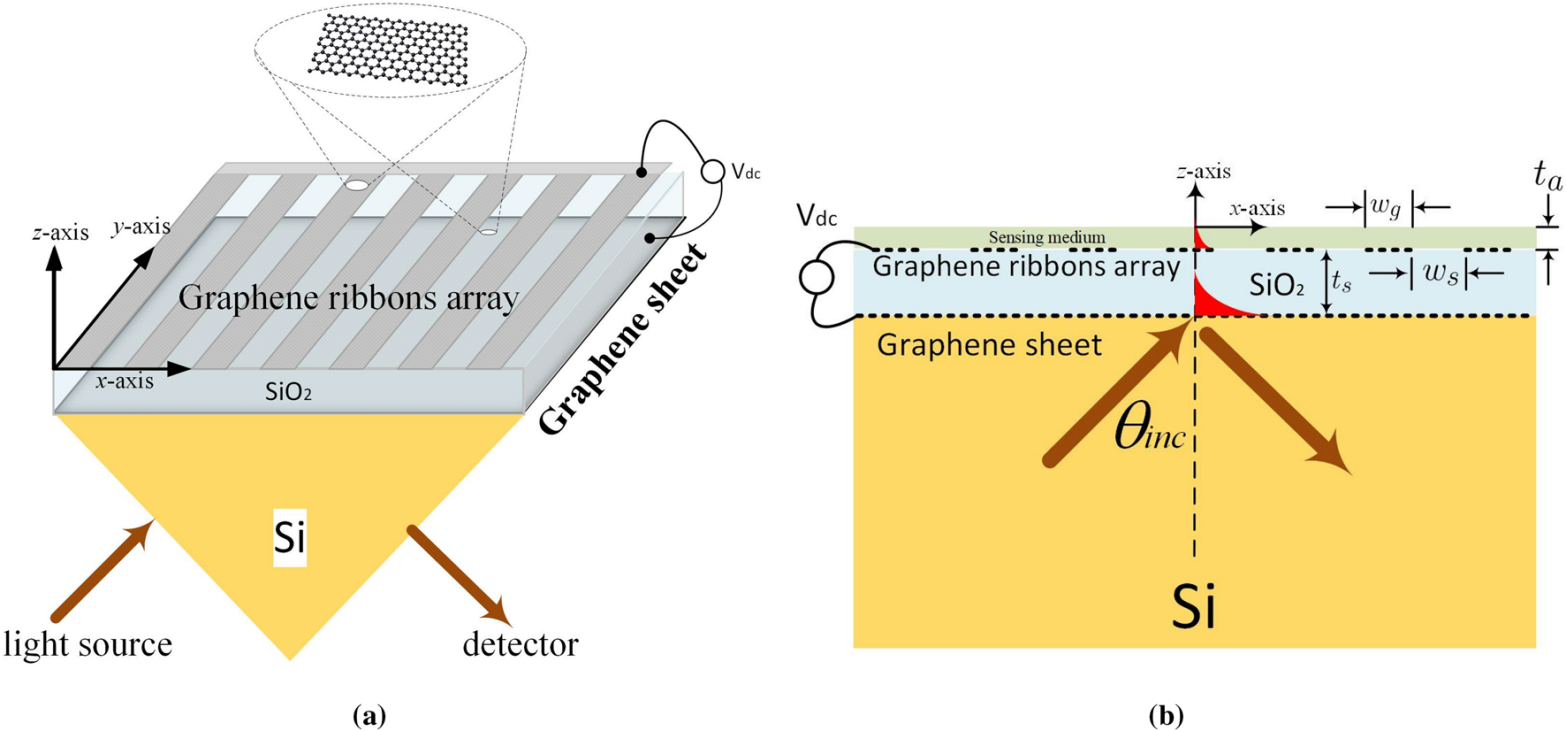
(**a**) Conceptual view of the evanescent wave biosensor, and (**b**) 2D model for electromagnetic full-wave simulation.

Figure  1a,b show the structure configuration of the biosensor under study; Fig. 1a is the conceptual view and Fig. 1b is the corresponding 2D electromagnetic model for full-wave simulation. The periodic graphene ribbons array (with period $d_x$) has ribbon width $w_g$ and slit width $w_s$ along the *x*-axis; they are deposited on a silica ($SiO_2$) having a frequency-dependent RI ($n_{SiO_2}+ik$)^30^ and thickness of $t_s$. Below the silica is a graphene sheet serving as an electrode; the self-biasing configuration including graphene ribbons array and graphene sheet is adopted^31,32^. Additionally, the graphene employed here is assumed to be zero thickness and its optical conductivity is a function of temperature (in Kelvin degree), carrier relaxation time ($\tau _c$) and chemical potential ($\mu _c$) that can be altered by the gate-controlled dc voltage applied between graphene sheet and ribbons array. Below the graphene electrode is a wedge-shaped silicon (Si) substrate with RI of $n_{Si}=3.4401$.

The single layer graphene inserted between the crystalline silicon and gate oxide has been successfully implemented in the structure of a graphene photodiode-oxide-semiconductor field effect transistor (PDOSFET) shown in Fig. 8 of^33^; the PDOSFET behaves like a normal n-channel MOSFET. The magnitude of the channel current of this phototransistor showed a logarithmic dependence on the illumination level; therefore, such a transistor can be a potential candidate for high dynamic range (HDR) image detection. The detail process of graphene transfer in fabricating the crystalline silicon-graphene-gate oxide structure can be found in Fig. 9 of^33^. Additionally, the graphene layer between Si and $SiO_2$ was proposed to develop a tunable graphene-based hybrid plasmonic modulator (GHPM)^34^. As was reported by the authors, this device is compatible with the conventional integrated circuits process. Regarding the issue of interaction between graphene and $SiO_2$ surface, in the paper of Novoselov et al.^35^, the electronic measurement showed that the single layer graphene on $SiO_2$ surface is possible to be doped chemically, enabling the spatially inhomogeneous doping effect of graphene supported by $SiO_2$ substrate. This can explain the intrinsic doping of a built-in chemical potential produced by charge transfer from the silica^29^.

The electromagnetic plane wave with TM-polarization (the magnetic field vector is along the *y*-axis) is normally incident upon the wedge surface shown in Fig. 1a. Here we assume that the structure dimension along the *y*-axis is much greater than the operating wavelength; the electric- and magnetic-fields have no variation along that direction. Therefore, a simplified 2D electromagnetic model depicted in Fig. 1b was developed to carry out the scattering analysis. Specifically, an electromagnetic plane wave is obliquely incident from the more dense medium (Si-substrate) to the less dense medium (silica) with incident angle $\theta _{inc}$, which is greater than the critical angle ($\theta _c=\sin ^{-1} (n_{SiO_2}/n_{Si})$). The total internal reflection takes place and the evanescent wave is excited in the silica layer. Moreover, such an evanescent wave^36^ exponentially decaying along the *z*-axis excites the SPR modes on the graphene ribbons. Notably, because that the period of graphene ribbons array ($d_x$) is much smaller than that of the operating wavelength under consideration, all the higher-order space harmonics are below cutoff; no transmit power is observed. The periodic array employed here, in fact, is to increase detection area. The structure dimensions of the biosensor employed in the numerical simulation are $w_g=w_s=20$ nm, $t_s=50$ nm, and $\theta _{inc}=30^\circ$, respectively. The thin silica layer used here is to reduce the required gate dc voltage; the maximum electric-field strength is smaller than 1*V*/*nm*, which is below the breakdown field in $SiO_2$ and graphene^37,38^.

**Figure 2:**
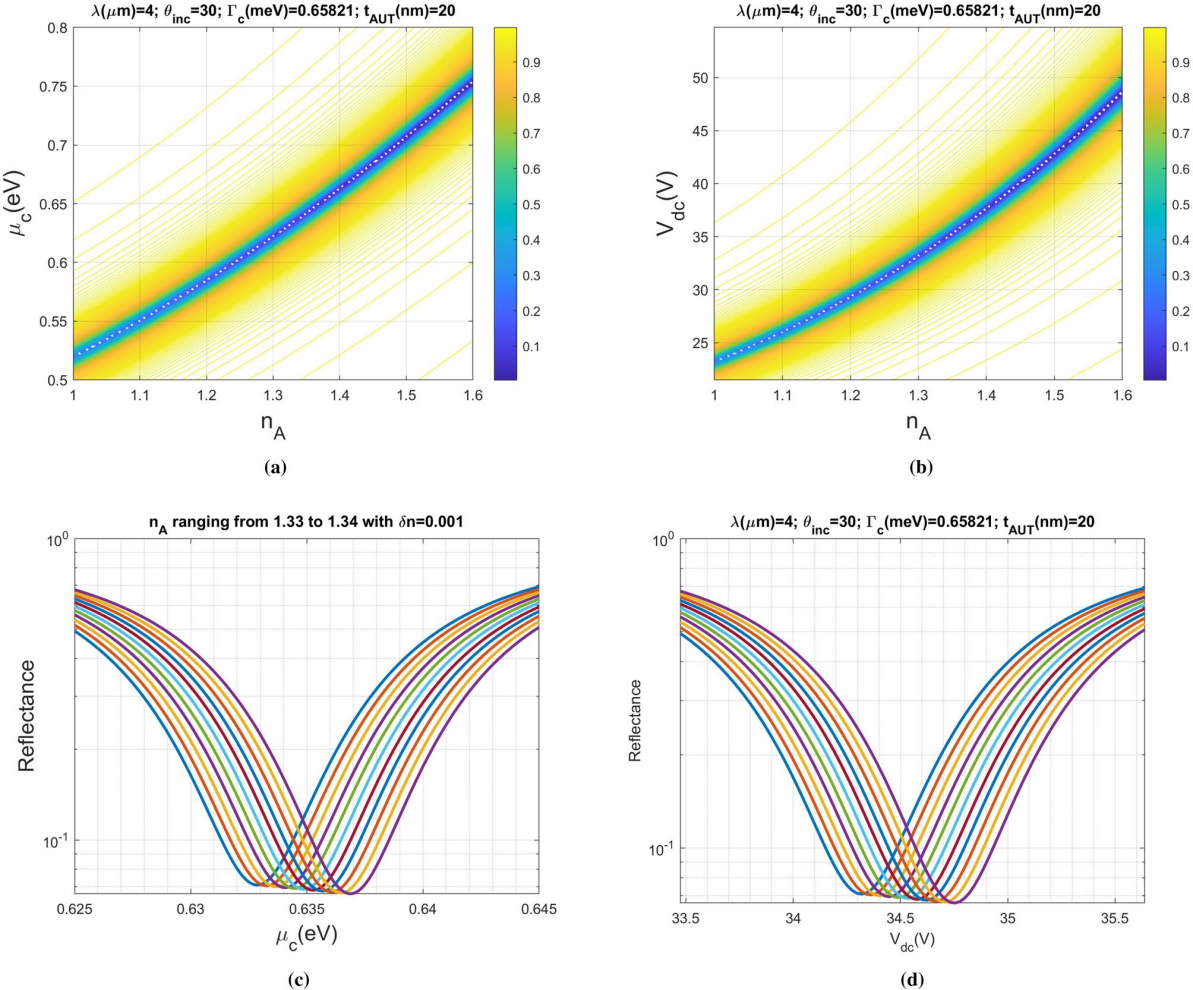
Contour of constant reflection coefficient for the biosensor excited by a light source operated at $\lambda =4\,\upmu$m with incident angle $\theta =30^\circ$, and sensing medium layer thickness 20 nm: (**a**) reflectance against chemical potential $\mu _c$ and $n_{A}$, (**b**) reflectance against applied gate voltage $V_{dc}$ and $n_{A}$, (**c**) reflectance versus chemical potential for various analyte RI, and (**d**) reflectance against applied gate voltage for various analyte RI. The refractive index of the analyte is ranging from $n_{A}=1.33$ to $n_{A}=1.34$ with step $\Delta n_{A}=0.001$.

Figure  2a,b shows the contour of constant reflection coefficient against analyte RI and graphene chemical potential (gate voltage). The relationship between gate voltage and chemical potential can be found in the later section. The analyte is modeled as a uniform dielectric layer with RI designated as $n_{A}$ and thickness of 20 nm. The operating wavelength of a TM-polarized electromagnetic wave (light) is $\lambda =4 \,\upmu$m, which can be generated by a quantum cascade (semiconductor) laser emitting in the mid-infrared spectrum^39^. The region in blue color exhibits low reflectivity, corresponding to the SPR condition where the incident power is dissipated mostly on the graphene ribbons array. As will become clear later on, there are plenty of SPR modes existed on the graphene ribbons; however, only the fundamental SPR mode is considered because of its strongest absorption compared with the other modes. Alternatively, the plot in Fig. 2a reveals that the SPR relates to both graphene chemical potential and analyte RI subject to given incident condition and structure parameters. Therefore, for a designated analyte RI, a unique chemical potential can be found in Fig. 2a to achieve SPR. From the perspective of experimental measurement, we may draw the SPR curve by measuring the reflectance against $\mu _c$ (via tuning $V_{dc}$) for a prescribed $n_{A}$. Once $n_{A}$ changes, the SPR curve shifts accordingly due to the position change of the reflectance dip. By detecting the SPR-curve shift, the variation in analyte can be observed. In Fig. 2b, the curve is piecewise linear; that is, we may define the sensitivity as $S(V_{dc})=\Delta V_{dc}/\Delta n_{A}$ in each subsection of $n_{A}$. In fact, parameter *S* is the slope of the curve with the unit of mV/RIU. Notably, instead of measuring SPR shift with respect to wavelength or incident angle scan in conventional approaches, such a scheme merely scans the gate voltage provided by a high-resolution digital-to-analog converter (DAC); therefore, it is more feasible and efficient than the commonly used methods.

We redraw the reflectance against chemical potential shown in Fig. 2c, and reflectance versus gate voltage depicted in Fig. 2d, respectively. The curves from left to right represent the reflectance of various refractive indices of analyte ranging from 1.33 to 1.34 with a step of $\Delta n_{A}=0.001$. As shown in Fig. 2c,d, the shift in SPR curve is clear enough to observe the change in RI. In Fig. 2d, the relationship between $\Delta V_{dc}$ and $\Delta n_{A}$ is approximately linear; the average voltage difference is $\langle \Delta V_{dc}\rangle =43.7187$ mv, which can be provided by a commercial available DAC.

**Figure 3:**
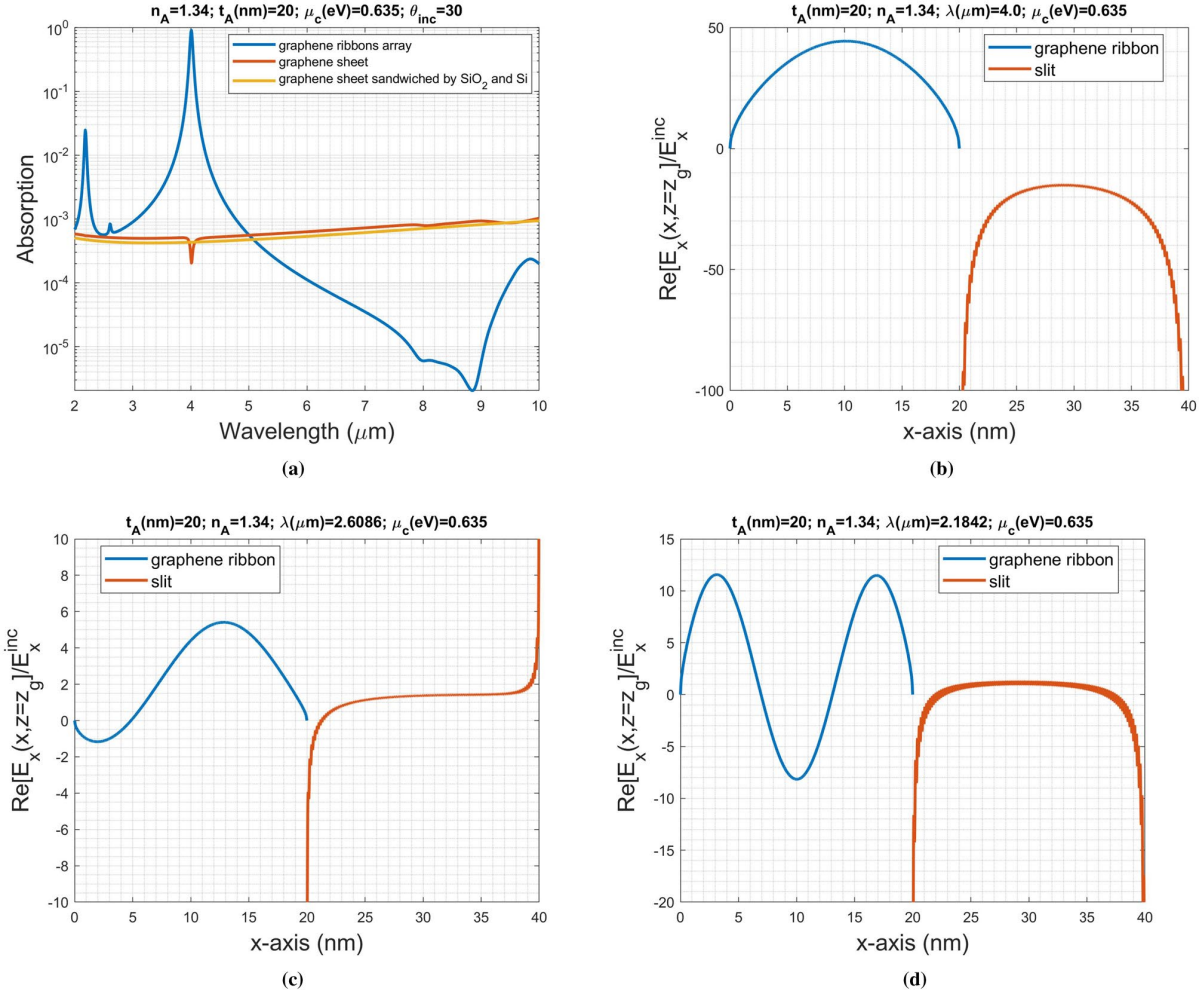
(**a**) Absorption against the incident wavelength for the periodic ribbons array (blue color) and graphene sheet (red color). The curve in orange color is that of the reference structure described in the main text. Real part of electric field component $E_x (x,z)$ over the unit cell for the three resonant modes at (**b**) wavelength equal to $4 \,\upmu$m, (**c**) wavelength equal to $2.6086 \,\upmu$m, and (**d**) wavelength equal to $2.1842 \,\upmu$m.

To understand the physical insight of wave process involved in the structure, the absorption against wavelength for the same biosensor employed in the previous example is calculated subject to an arbitrary analyte RI such as $n_{A}=1.34$. In Fig. 3a, the curve in blue color shows the absorption spectra of the periodic graphene ribbons array, while the red one depicts that on the graphene sheet served as an electrode. The curve in orange color is the absorptance of a reference structure consisting of a single graphene sheet sandwiched by the Si-substrate and silica (two semi-infinite mediums) without considering the graphene ribbons array but having the same evanescent-wave excitation. Obviously to see that the two curves with red and orange ones are close to each other; it indicates that the absorptance except for SPR regions, in general, can be predicted by that simple model. From the transmission-line network representation shown in Fig. S1 (Supplementary Information), the graphene sheet is modeled as a shunt admittance having $Y_g=\sigma _g$. The power absorption on the graphene sheet can be calculated by $P_{abs}=0.5 Re[VI^{\dagger }]=0.5|V|^2 Re[\sigma _g^{\dagger }]=0.5\sigma _{g,r}|V|^2$, where $\sigma _{g,r}$ is the real part of graphene optical conductivity ($\sigma _g=\sigma _{g,r}+i\sigma _{g,i}$) and *V* is the voltage induced on the graphene sheet.

Furthermore, the three wavelength-selective (resonant) absorption (in blue color) occurring at $\lambda =4.0\,\upmu$m, $\lambda =2.6086 \,\upmu$m, and $\lambda =2.1842 \,\upmu$m were observed. It is apparent to see that resonance indeed can enhance absorption. To explore the exact nature of resonant absorption on the periodic graphene ribbons array, we plot the real part of $E_x$ (normalized to the incident field) in the unit cell composed of graphene ribbon and slit region. Owing to the edge condition, its *x*-component current density ($J_x$) vanishes at ribbon edges ($x=0$ mm and $x=20$ nm), and so does its $E_x$ because of $J_x=\sigma _g E_x$. Figure  3b shows the first (fundamental) mode resonant at $\lambda =4.0\,\upmu$m; it is an even mode with symmetric field distribution with respect to the strip center. On the other hand, a small bump occurring at around $\lambda =2.6086 \,\upmu$m attributes to the second order mode with anti-symmetric field pattern (odd mode) depicted in Fig. 3c. The excitation of odd mode, in general, is caused by the incidence of an asymmetric $E_x$-field, for example, the oblique incidence in this case. Again, the third order mode resonant at $\lambda =2.1842 \,\upmu$m exhibits symmetry pattern (an even mode) shown in Fig. 3d. Such a graphene ribbon, in fact, can be regarded as a 1D nanoresonator. Moreover, let us look at the first mode with obvious absorption peak shown in Fig. 3b. The electric field ($E_x$) strength is considerably enhanced compared to the incident field, particularly at around the ribbon center and near both edges of slit. This can explain why even a small perturbation upon the location with strong $E_x$ can cause a significant change in the light scattering process, hence leading to the shift in SPR curve. In comparison with the enhanced evanescent-field uniformly distributed along the silicon surface^25^, although the fundamental SPR field, shown in Fig. 3b, around nanoribbon is enhanced, it is non-uniform along the *x*-axis. It is the reason why the guided-wave SPR (GWSPR) structure has a significant sensitivity enhancement. Nevertheless, due to the non-uniform evanescent field, the developed sensor can detect the redistribution of discrete analyte as shown in the later section.

Returning to Fig. 3b, the effective refractive index of the surface plasmon, defined as $n_{eff}=k_x/k_o$, at the first resonant mode can be determined from the $E_x$-distribution on the graphene ribbon. We employed the Matrix Pencil method^40^ by fitting a sum of exponential functions to the sampled data of $E_x$ at equal spacing from $x=0$ to $x=w_g$; that is, $E_x(x_k)=\sum _{j=1}^{P} A_j \exp {(B_j x_k)}$, where $x_k$ is assumed equally spaced. The two significant modes, which include forward- and backward-propagating ones, with $n_{eff}$ equal to $92.6+0.51i$ and $-92+0.4i$ were obtained, respectively. Notably, this resonance mode yields $k_x w_g\approx 0.92\pi$; it is distinct from the conventional resonator having $k_x w_g=\pi$.

### Performance evaluation: RI sensitivity and figure of merit

To evaluate the performance of this sensor, we calculate the RI sensitivity and figure of merit. The analyte under test is modeled as a uniform dielectric layer with refractive index $n_A$ and thickness of 8 nm. Here, four samples with $n_A=1.33$, $n_A=1.34$, $n_A=1.35$, and $n_A=1.36$ are considered. Table  1 shows the performance parameters of the biosensor. The first row is RI of analyte under test. The second row lists the sensitivities each of which evaluated in the vicinity of respective RI with an increment $\delta n_{A}=0.001$. The third row shows the full width at half maximum (FWHM) of the SPR curve against gate voltage for each case. Moreover, FOM (defined by FOM=S/FWHM) of each sample is listed in the fourth row. Apparently, the variation among those FOM is not obvious. It may be conjectured by the good linearity in the dip position change against $n_A$, shown in the Fig. S3 of the Supplementary Information.

The typical RI detection resolution is around $5\times 10^{-7}$ RIU for angular interrogation and $1\times 10^{-6}$ RIU for wavelength interrogation, respectively. Notably, the signal-to-noise ratio is the primary factor that dominates the instrument’s sensitivity resolution. Although the measurement uncertainties caused by mechanical noise can be eliminated by our approach (without moving parts), the temperature drifts, occurring evenly across all sensing schemes, is still an issue affecting the system performance. Therefore, the sensitivity resolution is dominated by the gate-voltage resolution in our approach.

Returning to Table  1, the 5th and 6th rows show the minimum gate voltage required for achieving the prescribed sensitivity for each RI. From implementation perspective, the gate voltage can be provided by a DAC. Notably, the output voltage, in fact, is discrete with a minimum step equal to $\delta V_{DAC}=V_{REF}/(2^N-1)$, where *N* is the bit number of a DAC and $V_{REF}$ is the reference voltage (full scale output). Since the maximum voltage needed to measure SPR curve is around 36*V* for $n_A=1.36$ (see Fig. S3 in Supplementary Information), the reference voltage is set to be $V_{REF}=36V$. The minimum voltage step is $\delta V_{DAC}=34.3\,\upmu$V for a 20-Bit DAC, $\delta V_{DAC}=8.6\,\upmu$V for a 22-Bit DAC, and $\delta V_{DAC}=2.1\,\upmu$V for a 24-Bit DAC, respectively. Apparently, to achieve a high resolution in RI sensing, a high resolution DAC is indispensable.

**Table 1:** Sensitivity (S), full width at half maximum (FWHM), and figure of merit (FOM) of the biosensor evaluated for the four samples.

$n_{A}$ (RIU)	1.33	1.34	1.35	1.36
S (mV/RIU)	36,401.1	40,676.5	40,918.2	41,160
FWHM (mV)	1667.1	1695	1723.4	1737.3
FOM (1/RIU)	21.84	24	23.74	23.69
$\delta V_{gate}$ (Resolution: $1\times 10^{-6}$ RIU)	36.4$\,\upmu$V	40.68 $\,\upmu$V	40.92 $\,\upmu$V	41.16 $\,\upmu$V
$\delta V_{gate}$ (Resolution: $5\times 10^{-7}$ RIU)	18.2$\,\upmu$V	20.34 $\,\upmu$V	20.46 $\,\upmu$V	20.58 $\,\upmu$V

### Sensing performance for protein monolayers

**Figure 4:**
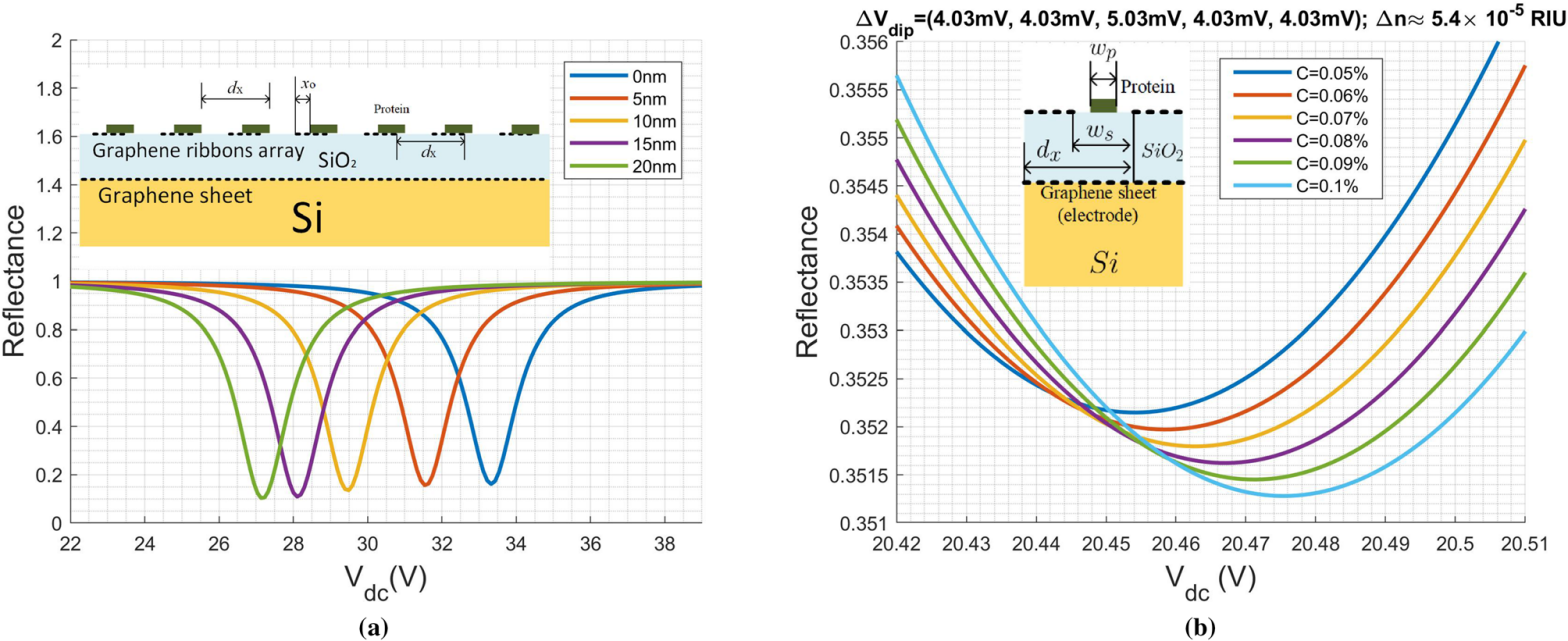
(**a**) Reflectance versus gate voltage for various (lateral) shift position ($x_o$) of the sensing targets, each with dimensions of $20\, \text{{nm}} \times 8\, \text{{nm}}$, modeled by a periodic dielectric layer with period $d_x$. (**b**) Reflectivity again applied gate voltage for various surface percentage coverage (or concentration: *C*).

In Fig. 4a, we replace the uniform dielectric layer by discrete dielectric bricks with $n_{A}=1.4393+i0.00040824$ at $\lambda = 4\, \upmu$m, which is the refractive index of protein monolayers^29^ (see supplementary information S4), in each unit cell (which can be regarded as a 1D periodic structure shown in the inset). The protein has width of 20 nm and thickness of 8 nm along the *x*- and *z*-axes, respectively. The distance between two adjacent proteins is fixed to $d_x$. The protein in each unit cell simultaneously changes its position along the *x*-direction (a lateral shift of the periodic layer) to mimic their redistribution. Figure 4a depicts the reflectivity versus gate voltage for various shift positions ($x_o$); for example, parameter $x_o=0 \,\text{{nm}}$ means that the protein placed on the graphene ribbon, while $x_o=20 \,\text{{nm}}$ represents that the protein is directly on the slit. Different lateral shift gives rise to different level of perturbation on the subwavelength ribbons array due to the non-uniform distribution of local field ($E_x$). The strongest absorption occurs when the proteins are directly placed on the slit having the strongest edge field. Interestingly, we may detect the redistribution of proteins by the shift in SPR curve (or change in absorption dip location).

In the second example, in each unit cell the protein monolayer is positioned at the center of slit, shown in the inset of Fig. 4b. We define the surface percentage coverage of protein as the ratio of $w_p$ to $w_s$ ($C=w_p/d_x$), where $w_p$ is the width of protein^41^. Parameter *C*, in fact, can also be regarded as the protein concentration. Here, $C=50\%$ corresponds to the result of green curve in Fig. 4a. As shown in Fig. 4b, the decrease in concentrations (*C*) from 0.1 to 0.05% with a step $0.01\%$ enables the SPR curve to shift toward low gate voltage. As shown in Supplementary Fig. S3, SPR curve moving toward lower dc voltage range represents the decreasing in $n_A$. Moreover, the voltage difference between the adjacent two dips are 4.03 mV, 4.03 mV, 5.03 mV, 4.03 mV, and 4.03 mV, respectively. The average change in RI is about $5.4\times 10^{-5}$RIU, which is estimated by averaging the RI of protein and air.

## Method of mathematical analysis

### Fourier modal method (FMM)

Vectorial electromagnetic fields satisfying the Maxwell Equations and boundary conditions in the 2D structure shown in Fig. 1b are considered to approximate the 3D one in Fig. 1a due to the assumption of large electrical length along the *y*-axis in practical manufacture; that is, the electric- and magnetic-fields are supposed to have no variation along that direction. Due to the periodicity along the *x*-axis (periodic graphene ribbons array), the electromagnetic fields in each uniform dielectric layer and semi-infinite medium are represented by Rayleigh expansions (or Floquet–Fourier series)^42–45^. Owing to the zero thickness approximation of the graphene grating, discontinuity in the tangential component of magnetic fields at the interface (surface of periodic graphene ribbons array) between two uniform mediums equals to the conduction current induced on the graphene strips array. The relationship between tangential electric and magnetic fields can be established via the graphene optical conductivity. Through the conventional mode-matching method, the scattering characteristics of the graphene ribbons array can be determined; however, the Gibbs phenomenon in Floquet–Fourier series due to the discontinuity of tangential electric field causes poor numerical convergence in scattering analysis. Specifically, as was reported^46,47^, traditional approach of rigorous coupled-wave analysis experienced a poor convergence in particular under the resonance of surface plasmon-polariton wave. Therefore, local basis functions inherently satisfying the field nature in graphene ribbon and slit regions were employed to modify the input–output relation of the periodic graphene ribbons array while the whole formulation can still fit into the standard procedure of Fourier modal method^12^. Some of the mathematical formulations together with the numerical convergence check (Fig. S1 in supplementary information), particularly at the resonance condition of SPPs, can be found in supplementary information.

### Graphene optical conductivity

Graphene conductivity ($\sigma _g=\sigma _{intra}+\sigma _{inter}$), having a close-form expression for the condition $\mid \mu _c \mid$ $\gg$ $k_B T$, consists of both the intraband ($\sigma _{intra}$) and interband ($\sigma _{inter}$) terms:^48^

1 $$\begin{aligned} \sigma _{intra}(\omega )= & {} \frac{2ie^2 k_B T}{\pi \hslash ^2(\omega +i\gamma )} \ln \left[ 2\cosh (\mu _c/2k_B T)\right] , \text{ and } \end{aligned}$$

2 $$\begin{aligned} \sigma _{inter}= & {} \frac{e^2}{4\hslash }\left\{ \frac{1}{2}+\frac{1}{\pi } \arctan \frac{\hslash (\omega +i\gamma )-2\mu _c}{2k_B T} -\frac{i}{2\pi }\ln \frac{[\hslash (\omega +i\gamma )+2\mu _c]^2}{[\hslash (\omega +i\gamma )-2\mu _c]^2+(2k_BT)^2} \right\} , \end{aligned}$$

where −*e* is the electron charge, $\hslash$ is the reduced Planck constant, $\gamma$ is a phenomenological carrier scattering rate ($\gamma =1/2\tau _c$, where $\tau _c$ is the carrier relaxation time), $\mu _c$ is the chemical potential, $k_B$ is Boltzmann’s constant, and *T* is the ambient temperature.

### The relationship between dc gate voltage and chemical energy

The carrier density ($n_s$) and the chemical potential ($\mu _c$) of the graphene layer are related through the equation given below:

3 $$\begin{aligned} n_s=\frac{2}{\pi \hslash ^2 v_f^2}\int _{0}^{\infty }\zeta [f_d(\zeta -\mu _c)-f_d(\zeta +\mu _c)] d\zeta \end{aligned}$$

where $f_d(\zeta )=(e^{(\zeta -\mu _c)/k_B T}+1)^{-1}$ is the Fermi-Dirac distribution; parameter $v_f$ is the Fermi velocity ($\approx 10^8 \text{{cm}} \cdot s^{-1}$ in graphene) and $\zeta$ is the energy. The self-biasing scheme is considered in Fig. 1a; therefore, the gate voltage can be determined by $V_{dc}=en_s/2C_{ox}$, where $C_{ox}=\varepsilon _s^{(dc)}\varepsilon _o/t_{S}$ is the gate capacitance of the silicon oxide in the unit of $F m^{-2}$; $\varepsilon _s^{(dc)}$ is the relative dielectric constant at dc (about 3.9 for $SiO_2$); the relation between $V_{dc}$ and $\mu _c$ can be approximated as $V_{dc}\approx \mu _c^2(e/2\pi C_{ox}\hslash ^2 v_f^2)$^49^.

## Parametric studies

In addition to the performance evaluation for the biosensor design described in the previous section, the effects of structure dimensions and graphene parameters on the reflectance have also been conducted and discussed in this section to provide adequate information for understanding the design criterion of such a biosensor.

**Figure 5:**
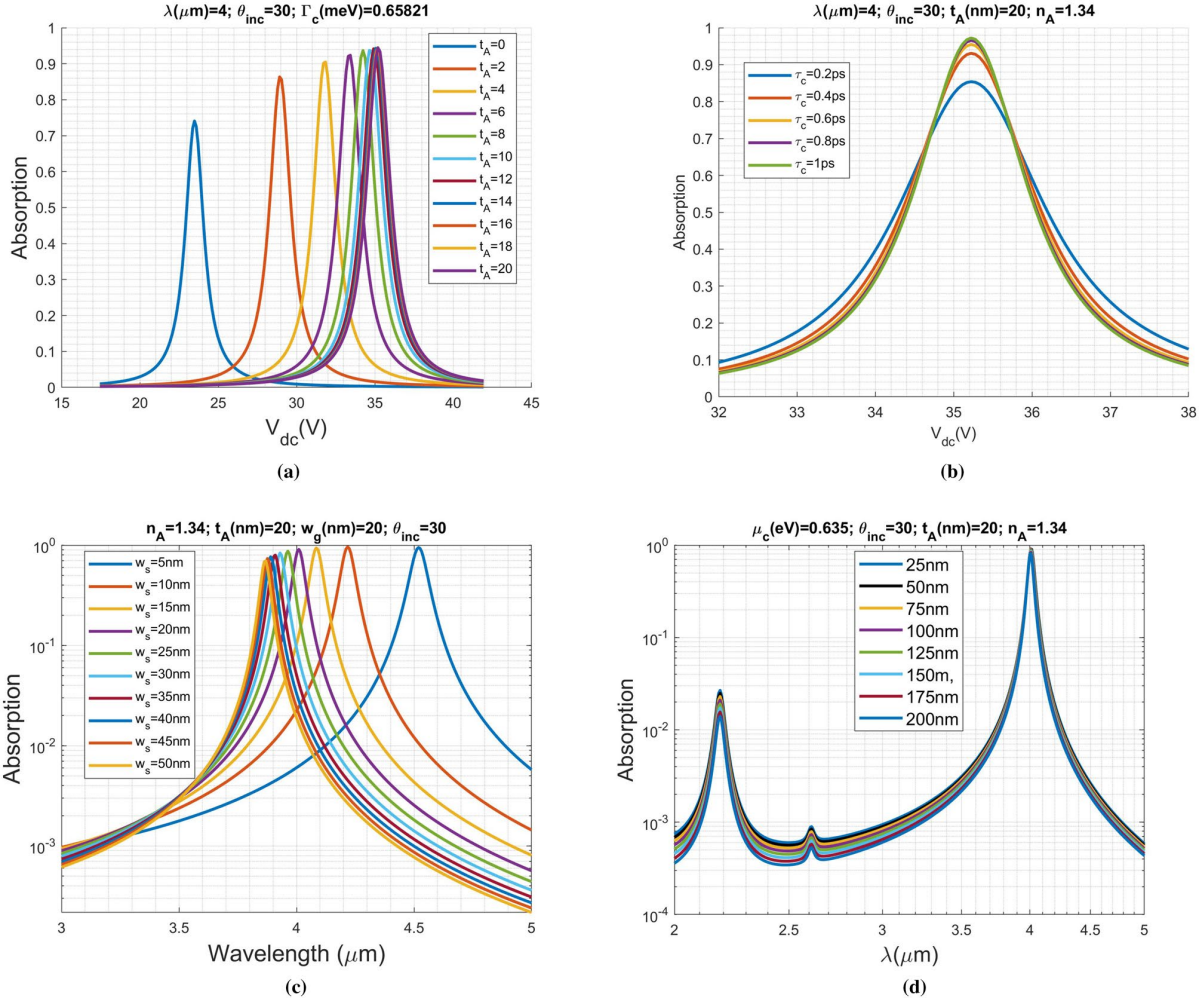
Parametric studies for the biosensor with typical structure dimensions including silica thickness, analyte thickness and slit width are 50 nm, 20 nm and 20 nm, respectively. The chemical potential and carrier relaxation time are 0.635 eV and 0.5 ps, respectively. The RI of analyte is $n_{A}=1.34$. (**a**) Variation of SPR curve (absorption efficiency of the graphene ribbons array) against gate voltage for various analyte thickness. (**b**) Variation of SPR curve against gate voltage for various carrier relaxation time ($\tau _c$). (**c**) Resonant absorption spectra for various slit widths and (**d**) Variation of SPR curve against wavelength for various silica thicknesses.

In the first example, we change the layer thickness of analyte to observe its influence on the SPR-curve. The thickness of silicon oxide is set to be 50 nm. The widths of graphene ribbon and slit both are 20 nm. The relaxation time is 0.5 ps. The incident angle and wavelength of the TM-polarized wave are $30^\circ$ and $4 \,\upmu$m, respectively. In Fig. 5a, we observe that the SPR curve is shifting toward high dc voltage in accordance with the increase in analyte thickness ($t_{A}$). Additionally, the absorption peak is increasing progressively. Interestingly, those SPR curves gradually coincide to one another as the layer thickness increases greater than 16 nm; that is, the deepest location that evanescent wave can penetrate is around 16 nm. Such a depth relates to the attenuation constant of the SPPs along the *z*-axis in the analyte. The rapid attenuation of the evanescent wave means that any object outside 16 nm will not affect the measurement.

As far as the sensitivity of a biosensor is concerned, the reflection dip for the frequency (wavelength)-selective property of the SPR curve is essential. A clear dip allows us to easily identify the RI change. In Fig. 5b, we progressively increase the graphene carrier relaxation time ($\tau _c$) to see the influence on absorption of incident light, where the analyte thickness is set to be 20 nm. The other parameters remain as given in the previous example. Apparently, the absorption is increasing in accordance with the increase in carrier relaxation time. Alternatively, the dielectric constant of a graphene sheet can be written as: $\varepsilon _{g}=1+i\sigma _g/\omega \varepsilon _0 \delta _g$, where $\delta _g$ is the graphene thickness roughly equal to 0.335nm. The real part of $\varepsilon$ is fixed at about $-\,106.76$ at the peak of absorption ($\mu _c \approx 0.64$ eV), supporting the SPPs on graphene. However, the imaginary part of $\varepsilon$ is increasing from around $-\,1.39$ to $-\,0.87$. Moreover, the quality-factor (Q) of such a graphene layer considered as a thin dielectric layer increases from 77 to 123; the increase and saturation in quality factor can also be qualitatively observed from the SPR curves shown in Fig. 5b in accordance with the increase in $\tau _c$.

In Fig. 5c, we evaluate the resonant absorption spectra for various slit widths ranging from 5 to 50 nm with a step of 5 nm, while the ribbon width is kept at 20 nm. The other structure dimensions remain as given in Fig. 5a. The chemical potential and carrier relaxation time are $\mu _c=0.635$ eV and $\tau _c=0.5$ ps, respectively. The analyte RI and thickness are $n_{A}=1.34$ and $t_{A}=20$ nm. Notably, only the fundamental mode of SPR was shown in this figure. The SPR is due to the resonant of SPPs along the ribbon-width direction and depends on the ribbon width^50^; however, it is apparent to see that the resonant wavelength is varying in accordance with the change in the slit width. Additionally, the line width of SPR curve is getting wide when the slit width is decreasing. This may be explained as the occurrence of strong mutual coupling between two adjacent ribbons for the case of narrow slit width. Moreover, the coupling is caused by the leakage in resonator^10^, reducing the quality factor (increase in line width) of SPR curve. Contrarily, the SPR curves gradually converges for $w_s$ greater than 40 nm; the graphene ribbons can thus be regarded as isolated ones with negligible coupling.

In Fig. 5d, we change the silica thickness to see the variation on absorption spectra. The resonant coupling to the graphene ribbons array is due to the evanescent wave in the silica. Moreover, the attenuation constants of evanescent wave in silica can be determined by $\alpha _z=k_o \sqrt{\varepsilon _{Si}\sin \theta _{inc}^2-\varepsilon _{SiO_2}}$, which is about $0.00154 \,\text{{nm}}^{-1}$ at $\lambda =4 \,\upmu$m and is around $0.0028 \,\text{{nm}}^{-1}$ at $\lambda =2.2 \,\upmu$m. For the silica thickness much smaller than a wavelength, the change in the impinging field on the graphene nanoribbons is insignificant at $\lambda =4 \,\upmu m$, while is distinguishable at $\lambda =2.2 \,\upmu$m, depicted in this figure.

## Conclusion

The computational design of an evanescent wave biosensor incorporating the SPR on periodic graphene ribbons array is carried out through the electromagnetic modeling and full-wave simulation. Different from the conventional approach by measuring the shift in SPR with respect to the operating wavelength or incident angle of a TM-polarized light, the self-biasing scheme by tuning the chemical potential of graphene (via modifying applied gate voltage) is implemented to track the SPR response. Moreover, due to ultrastrong $E_x$-field confinement on the surface of graphene ribbons array, particularly at around the slit edges, even a considerably small change in the RI of an analyte placed on its surface can be detected by observing the shift in SPR curve against gate-voltage scanning. Additionally, the strong evanescent wave, excited by the SPR, can effectively prevent the interaction with upper fluid, increasing the anti-interference ability. Interestingly to observe that such a non-metallic biosensor can also detect the redistribution (lateral shift) of the analyte modeled as a periodic array of discrete sensing mediums such as live viruses. This research develops a useful tool, which is based on Maxwell’s equations and material characteristics, for evaluating the performance of a graphene SPR-based biosensor. It facilitates the practical implementation and also provides a theoretical basis for understanding its underlying physics.

## Supplementary Information

Supplementary material 1

## References

[1] Soler M, Estevez MC, Cardenosa-Rubio M, Astua A, Lechuga LM (2020). How nanophotonic label-free biosensors can contribute to rapid and massive diagnostics of respiratory virus infections: Covid-19 case. ACS Sensors.

[2] Liu D (2015). High sensitivity refractive index sensor based on a tapered small core single-mode fiber structure. Opt. Lett..

[3] Quaranta G, Basset G, Martin OJF, Gallinet B (2018). Recent advances in resonant waveguide gratings. Laser Photonics Rev..

[4] Kim K, Yoon SJ, Kim D (2006). Nanowire-based enhancement of localized surface plasmon resonance for highly sensitive detection: a theoretical study. Opt. Express.

[5] Wu C (2012). Fano-resonant asymmetric metamaterials for ultrasensitive spectroscopy and identification of molecular monolayers. Nat. Mater..

[6] Rodrigo D, Tittl A, John-Herpin A, Limaj O, Altug H (2018). Self-similar multiresonant nanoantenna arrays for sensing from near- to mid-infrared. ACS Photonics.

[7] Salihoglu O, Balci S, Kocabas C (2012). Plasmon-polaritons on graphene-metal surface and their use in biosensors. Appl. Phys. Lette..

[8] Garcia de Abajo F. J (2014). Graphene plasmonics: Challenges and opportunities. ACS Photonics.

[9] Wang D (2019). Recent advances in surface plasmon resonance imaging sensors. Sensors.

[10] Nikitin AY, Guinea F, Garcia-Vidal FJ, Martin-Moreno L (2012). Surface plasmon enhanced absorption and suppressed transmission in periodic arrays of graphene ribbons. Phys. Rev. B.

[11] Carrasco E, Tamagnone M, Mosig JR, Low T, Perruisseau-Carrier J (2015). Gate-controlled mid-infrared light bending with aperiodic graphene nanoribbons array. Nanotechnology.

[12] Hwang R-B (2020). Highly improved convergence approach incorporating edge conditions for scattering analysis of graphene gratings. Sci. Rep..

[13] Zinenko TL, Matsushima A, Nosich AI (2020). Terahertz range resonances of metasurface formed by double-layer grating of microsize graphene strips inside dielectric slab. Proc. R. Soc. A Math. Phys. Eng. Sci..

[14] Homola J (2008). Surface plasmon resonance sensors for detection of chemical and biological species. Chem. Rev..

[15] Puiu M, Bala C (2016). Spr and spr imaging: Recent trends in developing nanodevices for detection and real-time monitoring of biomolecular events. Sensors.

[16] Zeng Y (2017). Recent advances in surface plasmon resonance imaging: Detection speed, sensitivity, and portability. Nanophotonics.

[17] Paliwal A, Tomar M, Gupta V (2018). Refractive index sensor using long-range surface plasmon resonance with prism coupler. Plasmonics.

[18] Arora P, Talker E, Mazurski N, Levy U (2018). Dispersion engineering with plasmonic nano structures for enhanced surface plasmon resonance sensing. Sci. Rep..

[19] Hutchinson AM (1995). Evanescent wave biosensors. real-time analysis of biomolecular interactions. Mol. Biotechnol..

[20] Homola J (2003). Present and future of surface plasmon resonance biosensors. Anal. Bioanal. Chem..

[21] Huertas CS, Calvo-Lozano O, Mitchell A, Lechuga LM (2019). Advanced evanescent-wave optical biosensors for the detection of nucleic acids: An analytic perspective. Front. Chem..

[22] Fang Y, Ferrie AM, Fontaine NH, Mauro J, Balakrishnan J (2006). Resonant waveguide grating biosensor for living cell sensing. Biophys. J..

[23] Liu PY (2016). Cell refractive index for cell biology and disease diagnosis: Past, present and future. Lab Chip.

[24] Zhou Y, Wang B, Guo Z, Wu X (2019). Guided mode resonance sensors with optimized figure of merit. Nanomaterials.

[25] Lahav A, Auslender M, Abdulhalim I (2008). Sensitivity enhancement of guided-wave surface-plasmon resonance sensors. Opt. Lett..

[26] Zhao Y (2020). Gese nanosheets modified surface plasmon resonance sensors for enhancing sensitivity. Nanophotonics.

[27] Vasić B, Isić G, Gajić R (2013). Localized surface plasmon resonances in graphene ribbon arrays for sensing of dielectric environment at infrared frequencies. J. Appl. Phys..

[28] Wu J (2014). Design of infrared surface plasmon resonance sensors based on graphene ribbon arrays. Opt. Laser Technol..

[29] Rodrigo D (2015). Mid-infrared plasmonic biosensing with graphene. Science.

[30] Kischkat J (2012). Mid-infrared optical properties of thin films of aluminum oxide, titanium dioxide, silicon dioxide, aluminum nitride, and silicon nitride. Appl. Opt..

[31] Gomez-Diaz JS (2015). Self-biased reconfigurable graphene stacks for terahertz plasmonics. Nat. Commun..

[32] Vakil A, Engheta N (2011). Transformation optics using graphene. Science.

[33] Tsai Y-Y, Kuo C-Y, Li B-C, Chiu P-W, Hsu KYJ (2020). A graphene polycrystalline silicon photodiode and its integration in a photodiode oxide semiconductor field effect transistor. Micromachines.

[34] Qu S, Ma C, Liu H (2017). Tunable graphene based hybrid plasmonic modulators for subwavelength confinement. Sci. Rep..

[35] Novoselov KS (2005). Two-dimensional gas of massless dirac fermions in graphene. Nature.

[36] Otto A (1968). Excitation of nonradiative surface plasma waves in silver by the method of frustrated total reflection. Z. für Phys. A Hadrons Nuclei.

[37] Sire C, Blonkowski S, Gordon MJ, Baron T (2007). Statistics of electrical breakdown field in hfo2 and sio2 films from millimeter to nanometer length scales. Appl. Phys. Lett..

[38] Fallahi A, Perruisseau-Carrier J (2012). Design of tunable biperiodic graphene metasurfaces. Phys. Rev. B.

[39] Razeghi M, Bandyopadhyay N, Bai Y, Lu Q, Slivken S (2013). Recent advances in mid infrared (3–5m) quantum cascade lasers. Opt. Mater. Express.

[40] Sarkar T, Pereira O (1995). Using the matrix pencil method to estimate the parameters of a sum of complex exponentials. IEEE Antennas Propag. Mag..

[41] Ye M, Crozier KB (2020). Metasurface with metallic nanoantennas and graphene nanoslits for sensing of protein monolayers and sub-monolayers. Opt. Express.

[42] Moharam MG, Gaylord TK (1983). Rigorous coupled-wave analysis of grating diffraction—e-mode polarization and losses. J. Opt. Soc. Am..

[43] Li L (1996). Formulation and comparison of two recursive matrix algorithms for modeling layered diffraction gratings. J. Opt. Soc. Am. A.

[44] Li L, Haggans CW (1993). Convergence of the coupled-wave method for metallic lamellar diffraction gratings. J. Opt. Soc. Am. A.

[45] Hwang R-BR (2012). Periodic Structures: Mode-Matching Approach and Applications in Electromagnetic Engineering.

[46] Fitio VM, Bobitski YV (2004). Resonance effects in a dielectric grating: Total absorption of electromagnetic waves by a dielectric grating on metal system. J. Opt. A Pure Appl. Opt..

[47] Fitio V, Yaremchuk I, Bendziak A, Bobitski Y (2020). Study of the resonance diffraction phenomena on gratings by the rigorous coupled wave method with modified equation system. J. Nano-Electron. Phys..

[48] Depine RA (2016). Electromagnetics of graphene. Graphene Opt. Electromagn. Solut. Canon. Probl..

[49] Mahdy MRC (2016). Electromagnetic metamaterial-inspired band gap and perfect transmission in semiconductor and graphene-based electronic and photonic structures. Eur. Phys. J. Plus.

[50] Nikitin AY, Low T, Martin-Moreno L (2014). Anomalous reflection phase of graphene plasmons and its influence on resonators. Phys. Rev. B.

