# Supplementary material for: A theoretical design of evanescent wave biosensors based on gate-controlled graphene surface plasmon resonance
Source: Sci Rep. 2021 Jan 21;11:1999. doi: 10.1038/s41598-021-81595-9 (PMC7820429; doi:10.1038/s41598-021-81595-9)
Supplement: Supplementary file 1 — Supplementary material 1 [file 41598_2021_81595_MOESM1_ESM.pdf]

Supplementary Information: A theoretical design of evanescent wave biosensors based on gate-controlled graphene surface plasmon resonance

Ruey-Bing Hwang
Institute of Communications Engineering
National Chiao Tung University
Hsinchu, Taiwan

S1: Method of mathematical analysis

Due to the periodicity along the x-axis (periodic graphene ribbons array), the electromagnetic fields in each uniform dielectric layer and semi-infinite medium are represented by Rayleigh expansions (or Floquet-Fourier series). The magnetic field of TM-polarization can be written as:

$$H_y(x,z) = \sum_{n=-\infty}^{+\infty} I_n(z)\psi_n(x), \tag{1}$$

where $\psi_n(x)$ is the nth space harmonic given as

$$\psi_n(x) = exp(ik_{x,n}x)/\sqrt{d_x}, \tag{2}$$

where the Fourier basis functions ($\{\psi_n\}$) form a complete set:

$$\langle \psi_m(x)|\psi_n(x)\rangle = \int_0^{dx} \psi_m^{\dagger}(x)\psi_n(x)dx = \delta_{mn}, \tag{3}$$

Parameter δ_{mn} is the Kronecker delta function, and $k_{xn} = k_o\sqrt{\varepsilon_{inc}}\sin\theta + n2\pi/d_x$; parameter ε_{inc} is the relative dielectric constant in the incident region, for instance, Si-substrate in this research; parameters m and n are integers running from negative- to positive-infinities; symbol " $\dagger$ " stands for complex conjugate.
The tangential electric field component along the x-axis is written as:

$$E_x(x,z) = \sum_{n=-\infty}^{+\infty} V_n(z)\psi_n(x). \tag{4}$$

Where the Fourier amplitudes $V_n(x)$ and $I_n(z)$, satisfying transmission line equations, are the voltage and current amplitudes of the nth space harmonic, respectively. Their general solutions can be written as follows [12].

$$V_n(z) = v_n^+ \exp(+ik_{zn}z) + v_n^- \exp(-ik_{zn}z), \tag{5}$$

$$I_n(z) = Y_n \left[v_n^+ \exp(+ik_{zn}z) - v_n^- \exp(-ik_{zn}z) \right], \tag{6}$$

with the propagation constant along the z-axis $k_{zn} = \sqrt{k_o^2\varepsilon_s - k_{xn}^2}$ and wave admittance $Y_n = \omega\varepsilon_o\varepsilon_s/k_{zn}$ in a medium with relative dielectric constant ε_s. Parameters v_n^+ and v_n^- usually are termed as the amplitudes of forward- and backward- propagating waves of the nth diffraction order, respectively. Furthermore, the voltage and current amplitudes of each space harmonic can be collected and put into vectors given below.

$$\underline{V}(z) = \exp(+i\mathbf{K_z}z)\underline{v}^+ + \exp(-i\mathbf{K_z}z)\underline{v}^-, \tag{7}$$

$$\underline{I}(z) = \mathbf{Y_c} \left\{ \exp(+i\mathbf{K_z}z)\underline{v}^+ - \exp(-i\mathbf{K_z}z)\underline{v}^- \right\}, \tag{8}$$

where $\mathbf{Y_c}$ is a diagonal matrix with the nth entry equal to Y_n. The nth element in column vectors $\underline{V}(z)$, $\underline{I}(z)$, $\underline{v}^+$, and $\underline{v}^-$ are $V_n(z)$, $I_n(z)$, v_n^+ and v_n^-, respectively. The nth element in the diagonal matrix $\exp(\pm i\mathbf{K_z}z)$ is $\exp(\pm ik_{zn}z)$.

Input-output relation of a uniform dielectric layer

Having the termination condition defined at the output of a uniform dielectric layer given below:

$$\underline{I}(z = t) = \mathbf{Y}_l\underline{V}(z = t), \tag{9}$$

where $\mathbf{Y}_l$, a full matrix in general, can be regarded as the load admittance matrix of the transmission lines between voltage ($\underline{V}$) and current ($\underline{I}$) waves at $z = t$. Therefore, the relationship between the unknown amplitudes a_n and b_n (now are expressed in terms of vectors $\underline{a}$ and $\underline{b}$) can be obtained through matrix operation, we obtain $\underline{b} = \mathbf{\Gamma}\underline{a}$.

$$\mathbf{\Gamma} = exp(i\mathbf{K_z}t)\mathbf{\Gamma}_l exp(i\mathbf{K_z}t), \tag{10}$$

with

$$\mathbf{\Gamma}_l = (\mathbf{Y}_c + \mathbf{Y}_l)^{-1}(\mathbf{Y}_c - \mathbf{Y}_l). \tag{11}$$

With the reflection matrix $\mathbf{\Gamma}$, the input admittance, which is defined by $\underline{I}(0) = \mathbf{Y}_{in}\underline{V}(0)$, can be obtained as:

$$\mathbf{Y}_{in} = \mathbf{Y}_c(\mathbf{I} - \mathbf{\Gamma})\left(\mathbf{I} + \mathbf{\Gamma}\right)^{-1}, \tag{12}$$

where $\mathbf{I}$ is the identity matrix.

Additionally, the voltage transfer matrix, which is defined as $\underline{V}(z = t) = \mathbf{T}_v(t;0)\underline{V}(0)$, is written below.

$$\mathbf{T}_v(t;0) = (\mathbf{I} + \mathbf{\Gamma}_l)exp(i\mathbf{k_z}t)(\mathbf{I} + \mathbf{\Gamma})^{-1} \tag{13}$$

Input-output relation of a periodic graphene ribbons array

The discontinuity of tangential magnetic fields at the interface (periodic graphene ribbons array at $z = 0$) between the lower and upper dielectric layers produces the conduction current flowing on the graphene surface, yielding the equation given below.

$$\underline{I}(0^-) - \underline{I}(0^+) = \mathbf{Y_g}\underline{V}(0), \tag{14}$$

where $\mathbf{Y_g}$ is a full matrix with each entry related to the graphene conductivity and structure dimensions [12]; the nth element in column vectors $\underline{I}(0^{\pm})$ and $\underline{V}(0)$ respectively are $I_n(0^{\pm})$ and $V_n(0)$; the symbol $\pm$ represents the upper- and lower-mediums sandwiching the graphene ribbons array.

The matrix $\mathbf{Y_g}$ in Equation 14 establishes a relationship between voltage- and current-wave amplitudes across the periodic graphene ribbons layer; it is the so-called admittance matrix in microwave engineering [39]. Having the input-output relation of the graphene ribbons array in Equation 14 and input-output relation of a uniform dielectric layer described previously, the scattering characteristic of the overall structure in Fig.1(b) can be calculated through the building block approach by cascading each of the transfer matrix (input-output relation of each layer including graphene ribbons array, graphene sheet and uniform dielectric medium). The detail mathematical formulation concerning the Fourier Modal Method can be found in the literature [39–42]. As was reported [43, 44], traditional approach of rigorous coupled-wave analysis experienced a poor convergence in particular under the resonance of surface plasmon-polariton wave. In this research, the local basis functions inherently satisfying the electromagnetic edge conditions in both the graphene ribbons and slits were employed [12] to effectively improve the numerical convergence and eliminate the Gibbs phenomenon at the discontinuities. Notably, the mathematical formulation employed in this research is rigorous; nevertheless, a finite truncation in the Fourier series is inevitable due to numerical computation. The mathematical formulation and computational results regarding the scattering analysis of a periodic graphene ribbons array have been successfully confirmed in the report.

More specifically, the sin-based local basis function vanishing at its both ends for any harmonic order is used to expand E_x over the graphene strip, which is given as

$$g_n(x) = \sqrt{\frac{2}{w_g}} \sin \frac{n\pi(x - x_1^{(g)})}{w_g}, \tag{15}$$

where $w_g = x_2^{(g)} - x_1^{(g)}$; the graphene strip belongs to the region of $[x_1^{(g)}, x_2^{(g)}]$; index n is ranging from 1 to N_g.

On the other hand, in the slit region, we have the singular basis functions with singularities at its two edges, which are commonly used to approximate the current parallel to the edges in a micro-strip line. They are expressed as follows:

$$s_n(x) = \sqrt{\frac{\gamma_n}{w_s}} \frac{\cos \frac{n\pi(x - x_1^{(s)})}{w_s}}{\sqrt{(w_s/2)^2 - (x - x_c^{(s)})^2}}, \tag{16}$$

where $x_c^{(s)} = (x_1^{(s)} + x_2^{(s)})/2$ and $w_s = x_2^{(s)} - x_1^{(s)}$; the slit is in the region of $[x_1^{(s)}, x_2^{(s)}]$. Parameter $\gamma_n = 2$ for $n = 0$ and $\gamma_n = 1$ for $n \neq 0$; index n runs from 0 to $N_s - 1$. Notably, the denominator

in $s_n(x)$ approximates $\sqrt{w_s}\sqrt{\rho}$ at the strip edge where $x = w_g + \rho$ ($x_1^{(s)} = w_g$ and $x_2^{(s)} = d_x$), which confirms the electric-field edge condition described previously.
Now, the admittance matrix in Equation 14 can be rewritten as:

$$[[Y_g]] = \left[\sigma_g[[G]] \quad [[0]]\right]\left[[[G]] \quad [[S]]\right]^{-1}, \tag{17}$$

Here, the sub-matrix $[[G]]$ and $[[S]]$ have the size N_{tot}-by-N_g and N_{tot}-by-N_s, respectively. Specifically, N_{tot}, N_g and N_s satisfy the relationship: $N_{tot} = N_g + N_s$. Moreover, the ratio between N_g and N_s equals to the ratio of w_g to w_s [12], therefore, we have $N_g = round[N_{tot} \cdot w_g/(w_g + w_s)]$ and $N_s = N_{tot} - N_g$; the operator $round[.]$ rounds a real number towards the nearest integer. In doing so, we have a square matrix $[[G]]$ $[[S]]$ of size N_{tot}-by-N_{tot}.

$$G_{mn} = \langle \psi_m(x)^\dagger | g_n(x) \rangle = \frac{-j}{\sqrt{2}}\sqrt{\frac{w_g}{d_x}} e^{jk_{xm}x_c^{(g)}} \cdot \left[e^{jn\pi/2} sinc(\alpha_{mn}^{+} w_g/2) + e^{-jn\pi/2} sinc(\alpha_{mn}^{-} w_g/2)\right], \tag{18}$$

$$S_{mn} = \langle \psi_m(x)^\dagger | s_n(x) \rangle = \frac{\pi}{2}\sqrt{\frac{\gamma_n}{w_s d_x}} e^{jk_{xm}x_c^{(s)}} \cdot \left[e^{jn\pi/2} J_o(\beta_{mn}^{+} w_s/2) + e^{-jn\pi/2} J_o(\beta_{mn}^{-} w_s/2)\right], \tag{19}$$

where $\beta_{mn}^{\pm} = k_{xm} \pm n\pi/w_s$ and $\alpha_{mn}^{\pm} = k_{xm} \pm n\pi/w_g$; function $J_o(\cdot)$ is the zero order Bessel function of the first kind; function $sinc(x)$ is the unnormalized sinc function defined as $sinc(x) = sin(x)/x$. Parameter n is the index of the local basis function.

Transmission-line network representation

The transmission-line network representation of the 2D model in Fig. 1B is shown in Fig. S1. Let's begin with the upper semi-infinite transmission line denoted as ″Air″. Because of un-termination, the intrinsic admittance matrix $\mathbf{Y}_a$ is regarded as the load admittance of the line denoted as ″Analyte″; $\mathbf{Y}_A$ is the intrinsic admittance of the Analyte layer. Through the input-output relation given in the previous section, the input admittance looking into the analyte layer can be obtained as $\mathbf{Y}_{in}^{(A)}$. Moreover, the admittance matrix ($\mathbf{Y}_g^{(RAs)}$) represents the input-output relation of the graphene ribbons array, shown in Equation (14). The input admittance looking into the graphene ribbons array equals to the shunt of $\mathbf{Y}_g^{(RAs)}$ and the input admittance $\mathbf{Y}_{in}^{(A)}$. This shunt admittance matrix is the load of the line termed as ″ Silica ″. Again, the input-output relation in the silica layer can be applied to determine its input admittance matrix denoted as $\mathbf{Y}_{in}^{(Silica)}$. Furthermore, the load admittance matrix of the line denoted as ″ Si-Substrate ″ equals to the shunt admittance of the graphene sheet and the input admittance looking into the silica layer equal to $\mathbf{Y}_g^{(S)} + \mathbf{Y}_{in}^{(Silica)}$. The reflection of a incident wave is due to the mismatch between characteristic admittance matrix $\mathbf{Y}_{Si}$ and the aforementioned load one. The reflection matrix is written below.

$$\mathbf{\Gamma} = (\mathbf{Y}_{Si} + \mathbf{Y}_g^{(S)} + \mathbf{Y}_{in}^{(Silica)})^{-1}(\mathbf{Y}_{Si} - \mathbf{Y}_g^{(S)} - \mathbf{Y}_{in}^{(Silica)}). \tag{20}$$

This completes the scattering analysis of the overall structure.

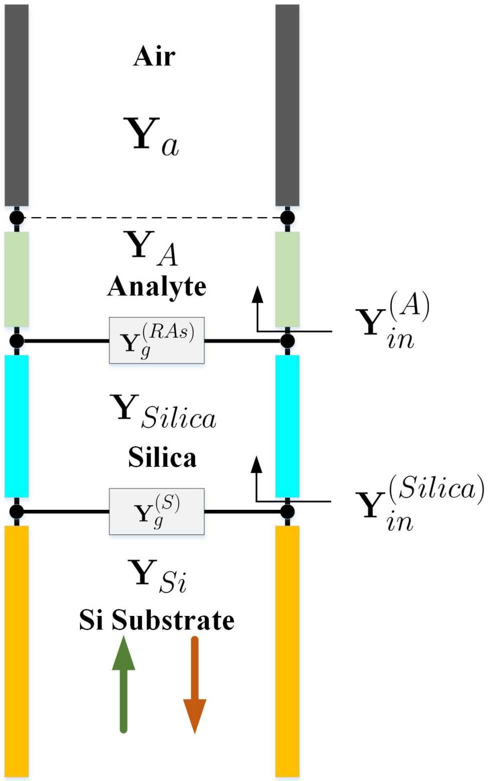

Figure S1: Transmission-line network representation of the structure shown in Fig. 1(b) of the 2D model of biosensor structure; $\mathbf{Y}_g^{(RAs)}$ (a full matrix) is the admittance matrix of the graphene ribbons array and $\mathbf{Y}_g^{(S)}$ (a diagonal matrix) is that of the graphene-sheet electrode.

Convergence check for the scattering analysis

In the Fourier Modal Method, the electric and magnetic fields both are expressed in terms of the Fourier series. However, due to the finite computation resources, we have to truncate the infinite to a finite one. Consequently, the index of Fourier components under truncation ranges from -N to +N with total truncated order equal to $2N + 1$. The convergence check for the absorption due to both graphene-sheet served as electrode (yellow curve) and periodic ribbons array (red curve), and reflectance (blue color) against the number of truncated order were carried out. Specifically, the convergence test is performed at the resonance condition of the SPPs. The excellent numerical convergence on the reflectivity and absorption, shown in Fig. S2, confirms the computation accuracy of the codes developed in this research.

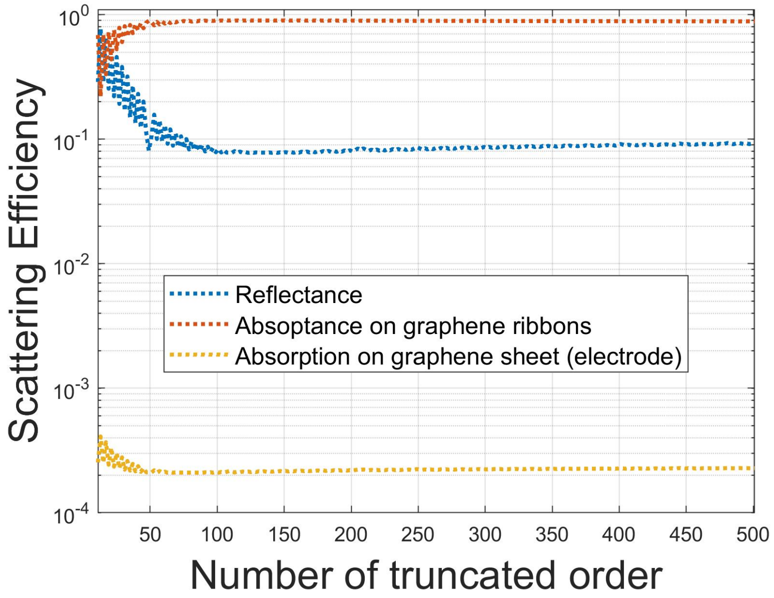

Figure S2: Scattering efficiency including the absorption efficiencies in both graphene sheet and graphene ribbons array and reflectance versus number of truncated order ($2N + 1$); the truncated space harmonic index is ranging from -N to +N: The structure dimensions of the biosensor are the same in Fig. 2. The analyte medium is modeled as a uniform dielectric layer with $n_A = 1.4393 + i0.00040824$ and $t_A = 8nm$; the operating wavelength is $4\mu m$; $\mu_c = 0.626eV$ and $\tau = 0.5ps$.

S2: Contour of constant reflectivity against analyte RI and gate voltage

The analyte under test is modeled as a uniform dielectric layer having refractive index denoted as n_A with thickness $t_A = 8nm$. The incident angle and wavelength are 30° and $\lambda = 4\mu m$, respectively. The structure parameters of the biosensor is the same as in Fig. 2. It is shown in Fig. S3 that the contour with smallest reflectivity (white color) is almost straight in the region where n_A is ranging from 1.33 to 1.36. This can explain why the figure of merit are consistent for the four cases with $n_A = 1.33$, $n_A = 1.34$, $n_A = 1.35$, and $n_A = 1.36$.

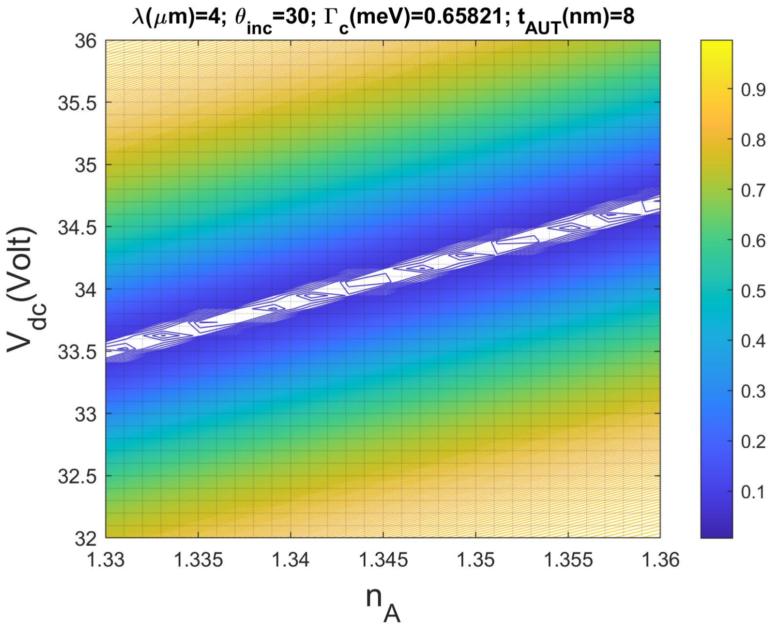

Figure S3: Contour of constant reflectivity against analyte RI (n_A) and applied gate voltage V_{dc}

S3: Protein permittivity

The protein permittivity can be modeled as a Lorentz series with two oscillating terms accounting for amide-I and amide-II bands, which is written below [29].

$$\epsilon_p(\omega) = n_\infty^2 + \frac{S_1^2}{(\omega_1^2 - \omega^2 - i\omega\gamma_1)} + \frac{S_2^2}{(\omega_2^2 - \omega^2 - i\omega\gamma_2)} \tag{21}$$

where the protein parameters are: $n_\infty^2 = 2.08$, $\omega_1 = 1668cm^{-1}$, $\omega_2 = 1532cm^{-1}$, $\gamma_1 = 78.1cm^{-1}$, $\gamma_2 = 101cm^{-1}$, $S_1 = 213cm^{-1}$, $S_2 = 200cm^{-1}$.
